# Nano-Drug Delivery Systems Based on Different Targeting Mechanisms in the Targeted Therapy of Colorectal Cancer

**DOI:** 10.3390/molecules27092981

**Published:** 2022-05-06

**Authors:** Ke Wang, Ruoyu Shen, Tingting Meng, Fuqiang Hu, Hong Yuan

**Affiliations:** College of Pharmaceutical Science, Zhejiang University, 866 Yuhangtang Road, Hangzhou 310058, China; wk1194229090@163.com (K.W.); 3150101386@zju.edu.cn (R.S.); mengtt@zju.edu.cn (T.M.); hufq@zju.edu.cn (F.H.)

**Keywords:** targeted therapy, colorectal cancer, nanoparticles, drug delivery systems

## Abstract

Colorectal cancer (CRC) is a usual digestive tract malignancy and the third main cause of cancer death around the world, with a high occurrence rate and mortality rate. Conventional therapies for CRC have certain side effects and restrictions. However, the exciting thing is that with the rapid development of nanotechnology, nanoparticles have gradually become more valuable drug delivery systems than traditional therapies because of their capacity to control drug release and target CRC. This also promotes the application of nano-drug targeted delivery systems in the therapy of CRC. Moreover, to make nanoparticles have a better colon targeting effect, many approaches have been used, including nanoparticles targeting CRC and in response to environmental signals. In this review, we focus on various targeting mechanisms of CRC-targeted nanoparticles and their latest research progress in the last three years, hoping to give researchers some inspiration on the design of CRC-targeted nanoparticles.

## 1. Introduction

Colorectal cancer (CRC), the third most common malignancy worldwide, forms in the colon or rectum. It is also the third most common cancer in men and the second in women separately. It is estimated that, by 2035, the number of CRC patients will reach 2.4 million and CRC will cause 1.3 million deaths worldwide [1,2].

According to the histopathological features, CRC can be divided into four distinct stages, which are also the basis of European Society for Medical Oncology (ESMO) guidelines to take appropriate treatments. In stage 0 of CRC, polyps are formed in the lining of colonic mucosa. Then, in stage I, polyps deteriorate into tumors and begin to migrate into the mucosa. The surgical resection of local tumors can achieve a good therapeutic effect without additional treatment in the above periods. There are many methods of operation, such as excision of polyps on the intestinal wall, excision of intestinal segments of tumors, or standardized excision, and finally reconnecting intestinal segments for ileostomy or colostomy. In stage II, cancer can spread beyond the colon, but it will not metastasize through lymph nodes at this stage. In stage III, the cancer cells spread to the colon wall and surrounding lymph nodes, but not to nearby organs. During these periods, the patients must be treated with both radiotherapy and chemotherapy. Fluoropyrimidine or combined with other chemotherapy drug is usually used to treat CRC. In addition, microsatellite instability status can be used to determine the survival time of patients with localized tumors, and patients with high microsatellite instability will have longer survival time. However, fluorouracil-based chemotherapy is not suitable for them and may even have adverse effects [3]. In stage IV, cancer accelerates to spread to other organs of the body [4]. At this time, it is not only necessary to remove the tumor through surgery, but also to kill the tumor cells through systemic chemotherapy or the combination of chemotherapy and biological targeted therapy. The biological targeted therapy of CRC uses bevacizumab, which can combine with the vascular endothelial growth factor produced by CRC cells to block tumor angiogenesis, cetuximab as well as panitumumab, which can bind to epidermal growth factor receptor (EGFR) overexpressed in CRC to prevent the growth of tumor cells.

As can be seen from the above, the survival rate of CRC patients can be increased if they are diagnosed as early as possible and treated accordingly, especially before CRC metastasis. Because the first step in the development of CRC is the formation of neoplastic polyps in the colonic mucosa, which are not very dangerous. However, because the specificity of early symptoms of CRC is not obvious and it is difficult to make a differential diagnosis, CRC patients are generally diagnosed in the advanced stage or when metastasis occurs [5].

Disappointingly, traditional therapies in this stage have certain side effects and limitations. For chemotherapy, due to poor physical and chemical properties, low bioavailability, and poor tissue selectivity of chemotherapeutic drugs, patients may suffer from many serious side effects [6,7,8]. Radiotherapy can cause serious DNA damage in patients sensitive to radiation damage, leading to the further development of tumors [9]. The side effect caused by receptor inhibitors is skin irritation [10]. Immunotherapy drugs cost a lot and have many side effects [11,12]. So, it is necessary to optimize the drug delivery to a specific target and minimize the side effects [13].

**Figure 1:**
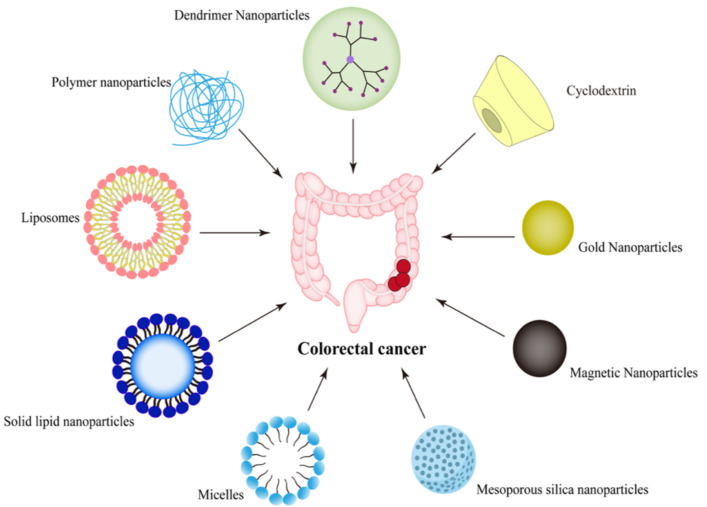
Types of nano-drug delivery systems targeting CRC.

**Figure 2:**
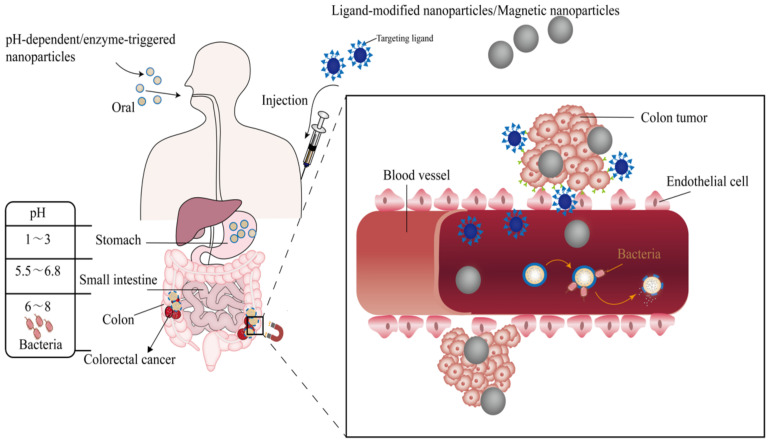
A schematic diagram of some representative targeting mechanism.

It is encouraging that, with the development of nanotechnology, there are many nano-drug delivery systems targeting CRC with high-efficiency anticancer, such as lipid nanoparticles, polymer nanoparticles, and liposomes, which can realize the delivery of chemotherapeutic drugs, genes, and vaccines. Various nano-drug delivery systems targeting CRC are presented in Figure 1. They can significantly improve the stability of drug in vivo, reduce the systemic toxicity of drug, and overcome drug resistance. Figure 2 shows some representative targeting mechanisms. This paper reviews the latest targeting mechanisms and prospects of nano-drug delivery systems for the treatment of CRC, with emphasis on the nanoparticles that can target the colon to further clarify the direction of future research and provide ideas for the design of nanoparticles for the better therapy of CRC.

## 2. Nano-Drug Delivery Systems Targeting CRC

### 2.1. Passive Targeting Nanoparticles

It is generally believed that the anatomy and pathophysiology of solid tumors differ from that of normal tissue. For example, solid tumors have a high blood vessel density to meet the nutrition and oxygen required by tumor cells growth. Furthermore, the tumor lacks functional lymphatic vessels and the gap between tumor endothelial cells is relatively large, which can extravasate or retain macromolecular drugs. The phenomenon that makes nanoparticles accumulate in tumor cells is called the enhanced permeability and retention (EPR) effect [14,15,16].

The discovery of this phenomenon has promoted the emergence of passive targeting nanoparticles, which can target tumors through the EPR effect. In research, oxaliplatin was encapsulated into N, O-carboxymethyl chitosan nanoparticles by ionic crosslinking, and resveratrol was encapsulated into them by emulsification crosslinking. The particle size of the former was about 190.0 nm and that of the latter was about 164.2 nm. The nanoparticles enhanced the solubility, stability, and EPR effect of oxaliplatin and resveratrol, showing stronger anti-CRC activity in subcutaneous tumor-bearing mice compared to the free drug [17].

However, according to research findings, nanoparticles smaller than 10 nm are filtered out by the kidneys, and particles larger than 100 nm are captured by the liver, so the ideal nanoparticles should be between 10 nm and 100 nm in size, which can circulate for a long time to increase the possibility of uptake by tumors [18]. Moreover, Anitha et al. [19] mentioned that the degree of vascularization in CRC is low. It can be seen that there are controversies about the EPR effect in CRC. In recent years, scientists have gradually realized that the tumor-targeting mediated by EPR is highly heterogeneous. Hence, it is necessary to enhance the targeting ability of nanoparticles based on EPR by combining them with other targeting mechanisms [20,21].

### 2.2. Active Targeting Nanoparticles

Ligand-modified nanoparticles can actively accumulate in the tumor through a ligand–receptor binding mechanism, thus delivering the drug to the target. These nanoparticles are called active targeting nanoparticles [22]. Cancer cells overexpress some types of receptors and secrete biomolecules that can promote cell proliferation of cancer and surrounding tissues through paracrine or autocrine pathways [23]. In studies, there are two main methods to combine ligands with nanoparticles: one is to modify them chemically during the synthesis of nanoparticles, and the other is to bind ligands with polymers before the synthesis of nanoparticles [24,25]. Ligand-modified nanoparticles can accumulate in the tumor through passive targeting, and then enter tumor cells through active targeting, resulting in a more selective and enhanced therapeutic effect.

In the recent three years, the active targeting design idea of nano-drug targeted delivery systems for CRC mainly adopt the receptor–ligand binding strategy, which involved many highly expressed receptors in CRC, such as folate receptor [26], EGFR [27], CD44, epithelial cell adhesion molecule (EpCAM) [28], CD133, αvβ3 integrin receptor, carcinoembryonic antigen [29], nucleolin [30], mannose receptor [31], hyaluronic acid receptor [32], *N*-acetyl-d-glucosamine [33], transferrin receptor [34], checkpoint kinase 2 [35], CXCR4+ [36], lipoprotein receptor-related protein-1 [37], MUC1 [38], NRP-1 [39], *P*-selectin [40], sigma-2 receptors [41], SSTRs [42], and glucocorticoid receptor [43]. The characteristics of these nanoparticles are shown in Table 1. Among them, the nanoparticles targeting EpCAM, folate receptor, epidermal growth factor, and CD44 were studied more.

#### 2.2.1. Nanoparticles Targeting EpCAM

Epithelial cell adhesion molecule (EpCAM) is a 314 amino acid and 39 kDa transmembrane glycoprotein that almost only exists on epithelial cells playing a role in adhering cells in normal cells [44,45]. As early as 1979, it has been reported as the main surface antigen of CRC [46], Later, scholars have found that overexpression of EpCAM also existed in human adenocarcinoma cells, and it could also carry out intercellular signal transduction [47].

In many kinds of research, to make nanoparticles target CRC after systemic administration, a DNA EpCAM aptamer (SYL3C) having a strong affinity with EpCAM protein and cancer cells expressing EpCAM was modified on nanoparticles. To reduce drug toxicity and improve therapeutic effect, Ge et al. [28] synthesized biological conjugates loaded with celastrol, which could be captured by CRC overexpressed with EpCAM. EpCAM aptamer, PEG, and dendrimers constituted the conjugates. The results showed that SW620 would undergo extensive apoptosis when exposed to biological conjugates. Moreover, in the xenograft mice and zebrafish models, the biological conjugate showed low toxicity. The dendrimer used in the study is composed of biocompatible components and has an excellent multivalent effect, which can improve the safety of chemotherapeutic drugs.

In another study, the author prepared a cationic liposome to deliver miR-139-5p. The main lipids contained in it are HSPC, DOTAP, Chol, and DSPE-PEG2000-COOH. Moreover, to make the nanoparticles target CRC, the surface of it was modified with EpCAM aptamer. It is found that nanoparticles had inhibitory effects on HCT cells, consistent with the experimental results of animal pharmacodynamics experiments. They also restrained the tumor growth of CRC mice injected subcutaneously with HCT8 [48].

In addition to the specific targets of CRC, some targets of other tumors are also expressed in CRC, which can be used as the targets of nano-drug delivery systems in the targeted therapy of CRC.

#### 2.2.2. Nanoparticles Targeting Folate Receptor

The folate receptor is a cell membrane-anchored protein, which is slightly expressed or absent in normal cells, but overexpressed in various malignant tumor cells, especially in CRC [22]. Therefore, it can be used as a target. Natural polymers chitosan and chondroitin sulfate have the advantages of biocompatibility and biodegradability. Soe et al. [26] prepared self-assembled nanoparticles using these two materials to incorporate the hydrophobic drug, bortezomib, and modified the nanoparticles to actively target folate receptors.

Folate was attached to DSPE-PEG and then coupled with nanoparticles. It was found that DSPE-PEG can not only promote blood circulation, but also improve core drug loading and stability. In the pharmacodynamic experiment of animal models, researchers found that nanoparticles had a strong inhibitory effect on the transplanted tumor and were almost non-toxic to other parts outside the tumor.

#### 2.2.3. Nanoparticles Targeting EGFR

The overexpression of epidermal growth factor receptor (EGFR) occurs in about one-third of epithelial malignancies. It can stimulate tumor growth, proliferation, promote angiogenesis, and promote cell invasion and metastasis. Therefore, the poor prognosis of CRC may have a great relationship with the overexpression of EGFR in CRC [49]. In order to solve this problem, targeted therapy using monoclonal antibodies to block biological pathways has been used to treat CRC.

The commonly used monoclonal antibody is cetuximab, which is used for CRC under FDA approval [50]. It can selectively target the EGFR to block signal transduction in vivo [49]. This characteristic also makes cetuximab widely used to modify nano-drug delivery systems, actively target CRC, and increase the accumulation in CRC [51].

Sankha Bhattacharya [27] formulated PLGA-PEG-coated nanoparticles to deliver anti-epidermal growth factor receptor-5-fluorouracil (5-FU), which could improve the pharmacodynamics and distribution of the drug in vivo. The Anti-EGFR mAb was bound to the polymeric nanoparticles by using m-maleimidobenzoyl-N hydroxysuccinimide ester to increase target specificity. On the other hand, polymeric nanoparticles consisting of PLGA and PEG can block opsonic action through the reticular epithelial system. These nanoparticles have great clinical value because their preparation methods are solvent emulsification and evaporation, which are simple and rapid [52].

Another attractive carrier is gold nanoparticles, which are commercially available and easily functionalized. Hallal et al. [53] found that AuNPs loaded with cetuximab had better EGFR endocytosis and could inhibit downriver signaling pathways compared with cetuximab and gold nanoparticles alone, which could inhibit cell proliferation and accelerate cell apoptosis. This finding is of great help in solving the problem of drug resistance in the treatment of EGFR monoclonal antibodies.

Moreover, many studies found that the therapeutic effect of monoclonal antibodies could be improved by combining McAb with chemotherapeutic drugs [54,55]. Chen et al. [56] constructed a hybrid nano delivery system to deliver 5-FU, including mesoporous silica nanoparticles (MSN), a supported lipid bilayer (SLB), and cetuximab. Furthermore, the SLB-MSN was functionalized with hydrophilic PEG, which made the nanoparticles more stable, circulate longer in the blood, and more likely to accumulate in the tumor through the EPR effect. Then, to enable the nanoparticles to actively target CRC, cetuximab was coupled to the PEG terminal in SLB-MSN. The SLB-MSN has the advantages of both MSN and liposome, showing significant biocompatibility. It can also encapsulate much drug, achieve controlled release, and its stability can be greatly improved.

#### 2.2.4. Nanoparticles Targeting CD44

CD44 is a transmembraneous glycoprotein that exists as an adhesion molecule on the cell surface and takes part in intercellular and cytomatrix interactions, as well as cell adhesion and migration [57]. The overexpression of CD44 in CRC cells is closely related to tumor metastasis [58,59]. Therefore, it is also a biomarker for CRC as well as a target. Hyaluronic acid (HA) is the main ligand of CD44, consisting of units of D-glucuronic acid and N-acetyl-D-glucosamine alternately. Especially, it is very popular with researchers, because of its non-toxic, excellent biocompatibility, and biodegradability [60].

Wang et al. [58] synthesized HA-decorated polydopamine nanoparticles to deliver chlorin e6 to the CRC. By coupling the HA with nanoparticles, the nanoparticles were easily internalized via endocytosis under the guidance of CD44. Moreover, chlorin e6 [61], the second-generation PS approved by FDA, and polydopamine, the photothermal agent with excellent light-thermal conversion efficiency were conjugated to promote the synergistic effect of photodynamic therapy and photothermal therapy by avoiding the toxicity induced by mental-ion. The result showed that the tumor growth was inhibited strongly in HCT-116 tumor mice, which also confirmed that the antitumor efficiency could be enhanced by combining the two treatments.

#### 2.2.5. Biomimetic Nano Delivery Systems

In addition to the ligand–receptor binding strategy, the nanoparticles can also actively target the tumor by bionic technology, which mainly uses biofilms to coat nanoparticles. This strategy can not only prevent nanoparticles from being identified by the immune system, but also make use of membrane proteins, glycoproteins, and homologous adhesion to make nanoparticles specifically accumulate in the tumor. Recently, the bionic cell membranes used in the reported nanoparticles are mainly erythrocyte membrane [62], cancer cell membrane [63], leukocyte membrane [64], and so on. Furthermore, to make the nanoparticles endowed with more properties, it is better to camouflage the nanoparticles with hybrid cell membranes.

Wang et al. [63] prepared Zn_1.25_Ga_1.5_Ge_0.25_O_4_: Cr^3+^, Yb^3+^, Er^3+^ persistent luminescence nanoparticles coated with mesoporous silica to deliver IR825 as well as irinotecan and encapsulated the nanoparticles with a cancer cell-macrophage hybrid membrane. The membrane made the nanoparticles actively target the tumor, then the nanoparticles continued to glow at the tumor, which could provide an accurate position for subsequent photothermal therapy. Moreover, after the CT26 tumor-bearing mice were treated with three months of nanoparticles and laser irradiation, the relative tumor volume of mice decreased significantly.

In a study, hollow long persistence luminescence nanomaterials loaded with cisplatin were synthesized and coated with a hybrid cell membrane, which was mainly composed of a customized erythrocyte membrane that could prevent nanoparticles from being recognized by the immune system and biofilm expressing PD-1 that gave nanoparticles targeting ability. Then, this team evaluated their efficacy through in vitro anti-cancer experiments and concluded that these nanoplatforms, which played the role of immunotherapy and chemotherapy, were efficient in CT26 tumor-bearing mice [65]. These studies indicated that bionic technology may be an effective method for tumor-targeted therapy.

## 3. Nano-Drug Delivery Systems in Response to Environmental Signals

### 3.1. Nanoparticles Based on Stimulus Response

Some nanoparticles can achieve precisely targeted therapy for CRC patients under the condition of stimulation, such as external magnetic field, temperature, reactive oxygen species (ROS), and near-infrared (NIR). In recent years, many pieces of research focused on magnetic hyperthermia. Magnetic nanoparticles were injected into the tumor and deposited under the action of an external magnetic field, and then give patients local radiofrequency hyperthermia [66].

In research, the complex of biological superparamagnetic chitosan-based nanocomposite was prepared to deliver SN-38 by combining with hydrophilic polymeric prodrug poly (l-glutamic acid)-SN-38, which showed obviously enhanced accumulation in CRC and more easily be internalized by cells with the assistance of a topical magnetic field. Moreover, magnetic nano complexes obtained a tumor inhibition ratio of up to 81% in the mice model of CRC xenografts [67].

More importantly, Dabaghi et al. [68] found that, compared with the use of magnetic hyperthermia individually or chemotherapy independently on the basis of magnetic nanoparticles loaded with 5-FU, the combination of the two was more effective in the treatment of CRC, and preferable therapeutic effects were also produced in mice model of CRC. It can be concluded that the magnetic nano complex system can boost tumor-targeted accumulation and improve the anti-colorectal cancer treatment effect.

Apart from the injection, rectal administration can also be used to treat CRC based on the difference between body temperature and external temperature. Xing et al. [69] prepared topotecan-loaded solid lipid nanoparticles (SLNs) and then encapsulated them into a thermo-sensitive aqueous gel to realize controlled release and to be low toxic. Because nanoparticles could maintain free flow below 30 °C and convert into gel form under physiological conditions, they are very convenient for rectal administration.

The construction of nanoparticles based on the response of the tumor environment is also the main strategy of targeted therapy. Considering that excessive ROS produced in the tumor promotes the transition from inflammation to cancer, Zhang et al. [70] synthesized a ROS-responsive and hydrogen peroxide-eliminating material by a cyclic polysaccharide, then used it to formulate functional nanoparticles loaded with irinotecan. Therefore, the nanoparticles can not only lighten oxidative stress and inflammatory response, but also release irinotecan under the simulation of the high level of ROS in the diseased colon. After oral administration of nanoparticles, the tumorigenesis and growth of colitis-induced CRC mice were expressively inhibited.

In addition to inducing photodynamic therapy for CRC [71], NIR can also be used as an external stimulus to stimulate the release of drug from nanoparticles at the target. Yadav et al. [72] designed a compact shell-crosslinked micelle to deliver indocyanine green (ICG) and doxorubicin (DOX), which could release the drug when stimulated by NIR, because under NIR irradiation, the ICG generated ROS which broke the structure of micelles by destroying the diselenide bond in the micelles, and then a large amount of DOX would be released. Additionally, Anugrah et al. [73] prepared a hydrogel composed of alginate to encapsulate ICG and DOX, whose diselenide bonds could be decomposed by the ROS generated by the ICG and then released DOX by gel-sol transformation under the NIR light. Therefore, NIR light-responsive drug delivery system can also be applied in the targeted therapy of CRC.

### 3.2. Oral Colon-Targeted Nano Delivery Systems

Nanomaterials will inevitably be recognized and swallowed by the immune system after intravenous injection, which will lead to adverse side reactions and reduce efficacy. In addition, the poor vascularization of CRC leads to a reduction in the number of nanoparticles by intravenous injection reaching the CRC and then limits the efficacy of nanoparticles [74].

Therefore, more and more scientists are committed to developing nano preparations that can be administered orally and still maintain the targeting of CRC. Furthermore, oral administration can improve patient compliance.

To achieve this goal, the colon-targeted drug delivery systems are supposed to prevent the drug from gastrointestinal degradation before the drug enters the colon, hence increasing the drug concentration in the tumor. In many reports about colon-targeted therapy as shown in Table 2, pH [75], time [76], or enzyme-responsive [77] nanoplatforms are designed for CRC.

#### 3.2.1. pH-Dependent Nano Platforms

Among various methods, the most focused approach is to develop a pH-dependent system. The gastrointestinal tract is structurally divided into gastro, small bowels, and intestinum crassum. In addition to the difference in physiological function, the pH of each part is also different. The pH of the stomach is 1~3, that of the small intestine increases to 5.5~6.8, which is close to the neutral environment, and the pH of the colon is 6~8 [81].

Inspired by enteric coating tablets, the researchers began to develop a nano-drug delivery system containing enteric-coated materials to make drug released in the colon [82]. At low pH values, these nanoparticles are intact, but at high pH values, due to the dissolution of the coating materials, the nanoparticles swell and adhere to the colon to release the drug on a special area. The two most widely used coating materials are Eudragit and polysaccharides.

Samprasit et al. [75] prepared nanoparticles that could adhere to the mucosa for the delivery of 𝛼-Mangostin to the colon against CRC, which could also stay in colon mucosa for a long time through adhesion. These nanoparticles were formed by chitosan and thiolated chitosan, the two were crossed by genipin, and the surface of nanoparticles was modified by Eudragit L100. There are many advantages of polymeric nanoparticles, such as encapsulating the drug, preventing drug degradation from various conditions, and improving the mucoadhesion and absorption within the GIT. However, they could not target the colon that was why the author modified the nanoparticles with Eudragit L100. Furthermore, it was mentioned that chitosan could not tolerate the acidity of the digestive tract after oral administration. The researcher in this article found that nanoparticles with chitosan as the main material and crosslinked with Eudragit S100 were inspiring vehicles that can specifically target the CRC and continuously release drug [83].

Pectin is a natural biopolymer extracted from plant polysaccharides, which acts as acid protection in the gastrointestinal environment [84]. Mohamed et al. [78] prepared SLNs coated with pectin and dry skim milk, which could release the encapsulated curcumin in the colon. Therefore, the oral bioavailability of curcumin was significantly enhanced.

In addition to Eudragit and polysaccharides, another paper [79] used beta-lactoglobulin as a carrier to keep irinotecan from being destroyed in the stomach and the drug was released in the small bowel. Moreover, the MTT assay showed that these nanocarriers are more toxic to HT-29 cancer cell lines and AGS than the free drug. With the discovery of more and more enteric-coated materials, targeted nano-drug delivery systems based on pH will be more and more used in the targeted therapy of CRC.

However, the efficacy of a pH-dependent system is still limited, mainly because the gastrointestinal tract varies greatly between different individuals. To deal with this problem, Taymouri et al. [76] prepared polymeric-coated capsules to deliver simvastatin (SIM) to the colon specifically, which was sensitive to both pH and time.

Firstly, for the purpose of improving the solubility of the drug, the author used the anti-solvent crystallization technology to prepare the nanosuspension of SIM and studied whether the nanosuspension could produce a better anticancer effect against HT-29 compared with free drug. Then, a capsule composed of ethyl cellulose and Eudragit S100 was developed, in which ethylcellulose had a controlled release performance and could produce time-dependent release and Eudragit S100 had pH-dependent solubility. Finally, the optimized nanosuspension was mixed with sodium dodecyl sulfate to be freeze-dried and loaded into the capsule. The findings showed that SIM was not released in the stomach, but in the colon. In addition, compared with free SIM, SIM nanoparticles significantly enhanced the cytotoxicity of HT-29.

There are some limitations of pH- or time-dependent nano-drug delivery systems. Firstly, pH-dependent nanoparticles may not fully target the colon because the colon (pH 6.8) is similar in pH to the small bowel (pH 7.4). Secondly, the uncertain time taken for gastrointestinal transit of the nanoplatforms makes time-dependent nanoparticles sometimes miss the targets [85]. Optimistically, designing nanoparticles that release drug dependent on colonic microbial degradation is another reliable strategy for the targeted therapy of CRC.

#### 3.2.2. Enzyme-Triggered Nanoparticles

There are more than 400 kinds of microbial flora in the colon, including two major categories of aerobic, such as Escherichia coli, and anaerobic, such as clostridium [86]. Moreover, some polysaccharides can only be degraded to smaller monosaccharides by the anaerobic microbiota of the colon, and then used by bacteria as an energy source, but cannot be digested by gastric and intestinal enzymes [87]. Hence, polysaccharides are of important function in the enzyme targeted therapy of CRC. They can not only control the location of drug release, but also are biodegradable and biocompatible natural polymers.

In a study, colonic enzyme-responsive dextran-based oligoester crosslinked nanoparticles were fabricated to deliver 5-FU. It was found in vitro release studies that the nanoparticles released 75% of the drug within 12 h of incubation with glucanase, but there was no drug release under the pH conditions of the stomach and small intestine [77]. Tiryaki et al. [85] prepared nanoparticles containing organic and inorganic materials, that is, silica aerogels were coated with dextran and dextran aldehyde. By coated with dextran and dextran aldehyde, drug loaded in the silica aerogel particles were released merely in the colon because the dextran was degraded by dextranase.

Apart from coating the nanoparticles, nanoparticles can also be incorporated into microparticles that are resistant to gastrointestinal enzymes. dos Santos et al. [80] synthesized chitosan nanoparticles loaded with 5-FU and then the nanoparticles were further encapsulated with microparticles made of degraded starch and pectin. Retrograde starch is a kind of modified starch that can resist enzyme degradation of the superior digestive tract. Additionally, their results showed that fewer nanoparticles were released from the microparticles than nanoparticles alone in the gastrointestinal lumen. It can be seen from these studies that polysaccharides are the key factor of enzyme-triggered colon targeted preparations.

## 4. Multifunctional Targeted Nanoparticles

In order to further improve the targeting of nano preparations, more and more scholars have designed nanoparticles with multiple targeting mechanisms. Rajpoot et al. [88] designed dual-targeted nanoparticles, which contained folate-modified SLNs and enteric polymer-coated alginate microspheres that encapsulated the nanoparticles. Because of the pH-responsive of enteric polymer, the enteric-coated microbeads could release drug in the colon region after oral administration. Additionally, the combination of folate enabled nanoparticles to target CRC. In another study, trackable near-infrared persistent luminescence mesoporous silica nanoparticles coated with lactobacillus reuteri biofilm (LRM) were prepared that could protect drug from being digested and reaching the colon. The LRM played an important role in the nanoparticles. It could not only lengthen the release time of 5-FU and protect the 5-FU in the stomach, but also endow the nanoparticles with the ability to actively target the colon because LRM could recognize CRC through some of its biological components, such as adhesin [89].

It is also desirable to design drug delivery systems based on multiple environmental signals response. Ma et al. [90] prepared a pH- and enzyme-dependent nano preparation to deliver chemotherapeutic drug. In the study, indomethacin, 5-FU, and curcumin were encapsulated, respectively, into Eudragit RS nanoparticles by nanoprecipitation and then incorporated in biphasic microcapsules composed of chitosan and hydroxypropyl methyl cellulose by aerosolization. At last, coating the microcapsules with enteric soluble Eudragit S100 could protect the microcapsules from being degraded in the stomach. Furthermore, the microcapsules were released until they reached the colon since the chitosan was decomposed by bacterial enzymes, which could make the drug accumulate in CRC.

## 5. Challenges of Nano-Drug Delivery Systems in CRC

Considering the high efficacy of nanoparticles in animal models of CRC, the clinical application of nanoparticles is promising. However, there are still some shortcomings of nano-drug delivery systems. The biggest challenge is how to produce nano-preparations on a large scale and evaluate their preclinical safety and effectiveness.

At present, the composition and structure of nanoplatforms for targeted therapy of CRC are becoming more and more complex, which makes the preparation process of nanoplatforms more complicated and difficult to be synthesized repeatedly. In addition, the physical and chemical properties of nanoplatforms should be controlled in the production process, which brings higher requirements to manufacturing units and increases the difficulty and cost of scale-up production. Microfluidic technology attracts the attention of researchers. Valencia et al. [91] made the self-assembly of lipid–polymer and lipid–quantum dots (QDs) realized by microfluidic technology, which prepared stable and uniform nanoparticles. On the other hand, the complex structure of nanoparticles also brings potential toxicity risks to patients, so it is necessary to select a model that is more similar to the pathogenesis of human CRC to evaluate their toxicity in the preclinical study [92].

Additionally, in clinical trials, it is found that nanoplatforms tend to reduce the toxicity of drug rather than improve efficacy [93]. According to the analysis of many scholars, most nanoparticles accumulate at tumors based on EPR effect, even actively targeted nano preparations, but EPR effect is more consistent among animals, while there are differences in EPR effect for CRC patients, which will affect the efficacy of nano preparations [94,95]. Therefore, personalized therapy is needed, that is, nanoparticles are used in patients with strong EPR effect to achieve better efficacy [96], or to use preparations that can accumulate in the tumor independent of EPR effect, such as temperature response-based hydrogel for local treatment of CRC.

Therefore, with the development of chemistry, biology, manufacturing, and other industries, nano-preparations will play a more important role in the targeted therapy of CRC.

## 6. Conclusions and Perspective

In the treatment of CRC, colon-targeted nano-drug delivery systems can change the distribution and release of drug in humans by accumulating in CRC, which achieves a better therapeutic effect and reduces side effects in comparison with traditional treatment methods. Therefore, how to design nanoparticles with high targeting ability has great significance for CRC. In the review, we mainly summarize and classify the colon-targeted nanoparticles from the perspective of targeting power, demonstrating the richness and creativity of nanoparticles targeting CRC in academic research. Due to the complex pathophysiological changes of CRC, multifunctional targeted nanoparticles may have greater targeting ability. The goal of these studies is to provide patients with more effective preparations. However, because of the differences between preclinical animal models and clinical CRC patients as well as the stability of nano preparations, the translation of targeted nano preparations from research to clinic is difficult, which are also the directions that we need to consider and focus on when conducting research.

## Figures and Tables

**Table 1:** Actively targeting nano delivery system for the treatment of colorectal cancer.

Ligand	Receptor	Nano Delivery System	Active Agents	Size (nm)	Charge (mv)	Administration Route	Ref.
Folate	Folate Receptor	Self-assembled nanoparticles	Bortezomib	196	28	IV	[26]
Anti-EGFR mAb	Epidermal Growth Factor Receptor	PLGA and PEG-based polymeric nanoparticles	5-Fluorouracil (5-FU)	252	−31	IV	[27]
EpCAM aptamer	Epithelial cell adhesion molecule	PAMAM dendrimers	Celastrol	300	−6	IV	[28]
SS-Fc	Carcinoembryonic antigen	PEGylated hollow mesoporous ruthenium nanoparticles	[Ru(bpy)2(tip)]^2+^, RBT	110	22	IV	[29]
AS1411 aptamer	Nucleolin	Silica nanoparticles coated with chitosan	AntimiR-21, doxorubicin (DOX)	87	16	IV	[30]
Mannose	Mannose receptor	Cyclodextrin-based host–guest complexes	Regorafenib	100	\	IV	[31]
Hyaluronic acid	RHAMM, CD44	Hyaluronic Acid–Doxorubicin nanoparticles	DOX	175	−5	IV	[32]
Wheat germ agglutinin	*N*-acetyl-d-glucosamine, sialic acid	PLGA nanoparticles	5-FU	156	−18	\	[33]
Transferrin	Transferrin receptor	Polymersomes	DOX	72	−2	IV	[34]
LRP-1 targeting peptide	Lipoprotein receptor-related protein-1	Human serum albumin nanoparticles	5-FU	208	−13	IV	[37]
MUC1 aptamer	MUC1	Mesenchymal-stem-cell-derived exosomes	DOX	50	−80	IV	[38]
Tumor-homing peptide tLyp-1	NRP-1	Nanoparticles	Paclitaxel, chlorin e6	107	−25	IV	[39]
Fucoidan	*P*-selectin	Nanoscale metal organic framework	Talazoparib, temozolomide	84	−18	IV	[40]
Anisamide	Sigma receptors	Lipidic core nanocapsules	Thymoquinone	217	−36	\	[41]
Dexamethasone	Glucocorticoid receptor	Cationic liposomes	ESC8, anti-Hsp90 plasmid	251	28	IV	[43]

**Table 2:** Selected examples of oral colon-targeted formulations.

Formulations	Ingredients	Targeting Strategy	Ref.
Polymeric nanoparticles	Chitosan and thiolated chitosan, Eudragit L100, genipin	pH responsive, mucoadhesiveness	[75]
Solid lipid nanoparticles	Pectin, skimmed milk powder, lipid	pH responsive	[78]
Beta-lactoglobulin nanoparticles	Beta-lactoglobulin	pH responsive	[79]
Polymeric coated capsule, nanosuspension	Ethyl cellulose, Eudragit S100	pH responsive, time-dependent	[76]
Polymeric nanoparticles	Dextran, bifunctional telechelic oligoester	Enzyme responsive	[77]
Microparticle	Chitosan, retrograded starch, pectin	Enzyme responsive	[80]

## Abbreviations

CRC	colorectal cancer
ESMO	European Society for Medical Oncology
EGFR	epithelial growth factor
EPR	enhanced permeability and retention
EpCAM	epithelial cell adhesion molecule
5-FU	5-fluorouracil
MSN	mesoporous silica nanoparticles
SLB	supported lipid bilayer
HA	hyaluronic acid
ROS	reactive oxygen species
NIR	near infrared
SLNs	solid lipid nanoparticles
ICG	indocyanine green
DOX	doxorubicin
SIM	simvastatin
LRM	lactobacillus reuteri biofilm
